# The Method Quality of Cross-Over Studies Involved in Cochrane Systematic Reviews

**DOI:** 10.1371/journal.pone.0120519

**Published:** 2015-04-13

**Authors:** Hong Ding, Guang Li Hu, Xue Yan Zheng, Qing Chen, Diane Erin Threapleton, Zeng Huan Zhou

**Affiliations:** 1 Department of Epidemiology, School of Public Health and Tropical Medicine, Southern Medical University, Guangdong, China; 2 Division of Epidemiology, School of Public Health and Primary Care, the Chinese University of Hong Kong, Hong Kong, China; University of British Columbia, CANADA

## Abstract

**Background:**

It is possible that cross-over studies included in current systematic reviews are being inadequately assessed, because the current risk of bias tools do not consider possible biases specific to cross-over design. We performed this study to evaluate whether this was being done in cross-over studies included in Cochrane Systematic Reviews (CSRs).

**Methods:**

We searched the Cochrane Library (up to 2013 issue 5) for CSRs that included at least one cross-over trial. Two authors independently undertook the study selection and data extraction. A random sample of the CSRs was selected and we evaluated whether the cross-over trials in these CSRs were assessed according to criteria suggested by the Cochrane handbook. In addition we reassessed the risk of bias of these cross-over trials by a checklist developed form the Cochrane handbook.

**Results:**

We identified 688 CSRs that included one or more cross-over studies. We chose a random sample of 60 CSRs and these included 139 cross-over studies. None of these CSRs undertook a risk of bias assessment specific for cross-over studies. In fact items specific for cross-over studies were seldom considered anywhere in quality assessment of these CSRs. When we reassessed the risk of bias, including the 3 items specific to cross-over trials, of these 139 studies, a low risk of bias was judged for appropriate cross-over design in 110(79%), carry-over effects in 48(34%) and for reporting data in all stages of the trial in 114(82%).Assessment of biases in cross-over trials could affect the GRADE assessment of a review’s findings.

**Conclusion:**

The current Cochrane risk of bias tool is not adequate to assess cross-over studies. Items specific to cross-over trials leading to potential risk of bias are generally neglected in CSRs. A proposed check list for the evaluation of cross-over trials is provided.

## Introduction

A cross-over trial is one in which subjects are given sequences of treatments with the object of studying differences between individual treatments (or sub-sequences of treatments) [1]. Compared with parallel group trials, cross-over studies trials have some advantages [2–4]: Firstly, every participant included in cross-over studies acts as his or her own control, enabling zero between-participant variation; secondly, the same statistical power may be obtained with fewer participants; thirdly, since all participants receive all interventions, participants receive equal benefit from the interventions. Cross-over studies are suitable for evaluating interventions with a temporary effect in the treatment of stable, chronic conditions [1,3]. Cross-over studies are also extremely popular for the study of new and developmental drugs [2,5]and this popularity means they are frequently included in systematic reviews.

Quality assessment is one of the most important aspects of systematic review as it determines the credibility of the conclusions [3].A variety of quality assessment standards, such as the Cochrane Collaboration’s tool for assessing the risk of bias [3], and the Jadad scale [6] are applied in systematic reviews. Quality assessment tools generally consider sequence generation, allocation concealment, blinding and so on, which are not fully appropriate for cross-over studies where principally arises from inappropriate cross-over design, carry-over effect or biased data reporting among other factors. [3]. Nevertheless, these standards are still widely used to assess cross-over studies in systematic reviews and although there is no specific quality assessment standard for cross-over studies, the Cochrane handbook offers some suggestions. It recommends that four questions should be asked [3]: 1) was the use of a cross-over design appropriate? 2) Is it clear that the order of receiving treatments was randomized? 3) Can it be assumed that the trial was not biased from a carry-over effect? 4) Are unbiased data available? In addition to these four questions, other issues, such as blinding and loss of follow up, also affect the quality of cross-over studies [1].

The aim of this study was to evaluate the limitations of current quality assessments in cross-over studies. As such, in this study, we selected a sample of Cochrane Systematic Reviews (CSRs) as the research objects and re-assessed the risk of bias in included cross-over studies.

## Methods

### Search strategy and study selection

We searched the Cochrane Library (2013 issue 10) for CSRs which included cross-over studies with the following keywords: “crossover”, “cross-over”, “changeover”, “change-over”, “N-of-1”, “N of 1”, among others. Two authors independently read the titles and full texts, and excluded CSRs that did not include any cross-over studies in addition to excluding CSR protocols, methods studies or diagnostic test accuracy reviews. Disagreement was resolved by discussing with a third author. Of the included studies, we sampled 60 CSRs to examine in details. Sampling was carried out using a computer-generated random number list. Firstly, we obtained a list of random numbers using Microsoft Excel and assigned each potentially eligible CSR a random number. We then sorted the CSRs lists on random number with an order from small to large. The first 60 studies were included for further evaluation and each cross-over study included in these 60 CSRs was included for further evaluation.

### Data abstraction

Data abstraction was conducted independently by two authors using pri-designed forms for both CSRs and cross-over studies. The data abstracted from CSRs included study title, authors, publication date, quality assessment information (assessment tools, items, and the results). From cross-over studies, basic information (study title, authors, publication date) was extracted in addition to details of methodological quality. The data extracted by two authors were cross-checked and the discrepancies were resolved by discussion with a third author.

### Bias assessment in included cross-over studies

Based on the Cochrane handbook and expert comments, we applied nine standard items to evaluate the risk of bias in: (1) appropriate cross-over design; (2) the randomized order of receiving treatment; (3) carry-over effects; (4) unbiased data; (5) allocation concealment; (6) blinding; (7) incomplete outcome data; (8) selective outcome reporting; and (9) other biases. All the items were judged as high, unclear, or low risk of bias based on the study methods reported in the original article (see Table 1). When necessary, we extracted supplemental information from the quality assessment in the CSRs. The quality assessment was undertaken independently by two authors, with discrepancies resolved by discussion. Results of study quality assessment were summarized using a narrative approach. Frequency data are presented as numbers and percentages.

**Table 1:** Quality assessment standard for a cross-over study.

Item	Description	Scoring	
*1. Appropriate cross-over design*	Three points are considered: (1) the condition of the patients should be chronic and stable; (2) the intervention should not provide permanent change, but rather temporary relief; (3) the effect of the first intervention should not last into the second treatment period.		Low: all the three points are absolutely correct;
			Unclear: it hard to judge because some information was missing or ambiguous;
			High: one or more points are incorrect.
*2. Randomized treatment order*	The order of receiving treatments should be randomized adequately.		Low: the method is appropriate and clearly described;
			Unclear: it is described as “randomized”, but it is hard to judge whether the implementation was adequate because some information (method, etc.) was not provided;
			High: the method is inappropriate, or no randomization is applied.
*3. Carry-over effect*	The authors should evaluate the carry-over effect and provide relevant information clearly.		Low: carry-over effect was evaluated and the results showed no carry-over effect;
			Unclear: carry-over effect was not evaluated, and it is hard for evaluators to judge;
			High: carry-over effect was evaluated and the results showed apparent carry-over effect, or indicated evidently from some other provided information.
*4. Unbiased data*	That only first-period data are available is considered a risk of bias.		Low: data for every period are provided;
			Unclear: data are unavailable for part of outcomes, or only analytical results are provided and it is hard to judge whether the results are analyzed based only on data from the first-period or every period.
			High: only first-period data are available.
*5. Allocation concealment*	The study should apply appropriate approaches to ensure the allocation sequence is concealed.		Low: allocation sequence was concealed adequately by appropriate methods;
			Unclear: concealment approaches were not described, or relevant information was ambiguous;
			High: no approaches to allocation concealment were used, or concealed inadequately.
*6. Blinding*	The study should apply a proper blinding method to prevent performance and detection bias. Those involved in blinding (participants, doctors, measurers, or analysts) depends on the particularity of the studies.		Low: appropriate blinding method was applied; No blinding, but the outcome and the outcome measurement are not likely to be influenced by lack of blinding;
			Unclear: relevant information was not provided;
			High: no blinding method was applied, or applied incorrectly, or ineffectively, which very likely affected the outcome.
*7. Incomplete outcome data*	The authors should provide relevant information about the completeness of outcome data, including the level of incompleteness, reasons, and analytic method to tackle these data shortcomings, etc.		Low: no missing outcome data, or the reason is acceptable, or missing outcome data were appropriate analyzed;
			Unclear: it is hard to judge because some information was not provided;
			High: missing outcome data existed and the reasons were unacceptable, and the analytic method was inappropriate.
8. Selective outcome reporting	The authors should report all the outcomes fully. Selective reporting of part of outcomes or data for an outcome or subsets of the data or analyses using the same data and etc. should be avoided.		Low: fully reported;
			Unclear: it is hard to judge due to the unavailability of some original information;
			High: the reports of the study suggest a high risk of selective outcome reporting.
9. Other bias	Any other potential risk of bias that may affect the quality of cross-over studies.		Low: the study is apparently free of other problems;
			Unclear: whether certain problems existed and led to a risk of bias is uncertain;
			High: high risk of bias existed due to evident problems.

*NOTE*: the standard was summarized from the Cochrane Collaboration’s tool for assessing risk of bias and the Cochrane handbook`s suggestions for assessing risk of bias in cross-over studies. The assessment of some items, especially items 5–8, are almost the same as that described in Cochrane Collaboration’s tool for assessing the risk of bias.

## Results

**Fig 1:**
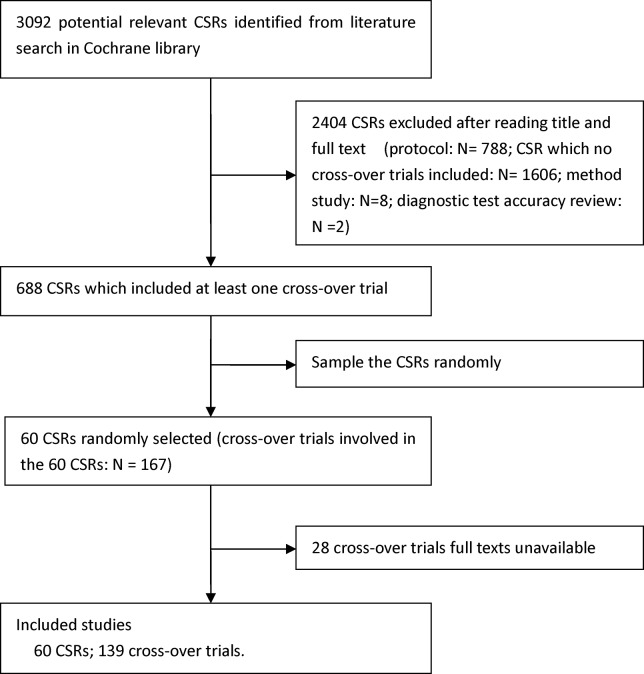
Flow chart for selection of studies.

The literature search of the Cochrane library yielded 3092 citations, of which 688 CSRs that involved at least one cross-over studies were found. We randomly sampled 60 CSRs (see S1 Appendix) and from these 139 cross-over studies were obtained and re-evaluated (see Fig 1). We screened the full text of the 688 CSRs, only one followed the Cochrane handbook’s suggestions for quality assessment for cross-over studies [6]. This CSR was not included in the 60 reviews that were sampled for the present study (see S1 PRISMA Checklist).

More than 6 different quality assessment tools were applied in the 60 CSRs. Twenty six reviews applied the Cochrane Collaboration’s tool for assessing the risk of bias; 15 CSRs applied the Jadad Scale; 3 CSRs applied specialized quality assessment tools designed by Cochrane review groups (Cochrane Bone, Joint and Muscle Trauma Group methodological quality assessment tool [7],The Cochrane Back Review Group methodological criteria list [8], and The Neonatal Cochrane Review Group criteria [9]); One CSR applied quality criteria developed by the US Preventative Services Task Force [10]; and the quality assessment tools applied in 17 CSRs were of unknown origin or were created by the review authors. In addition, 2 CSRs applied both the Cochrane handbook standard and the Jadad Scale [11,12]. Though several CSRs [13–15] considered “wash out” or “carry-over effect” in cross-over studies, none of the sampled CSRs comprehensively followed the suggestions from Cochrane handbook to formally assess the potential risk of bias.

### Included cross-over studies risk of bias assessments

**Fig 2:**
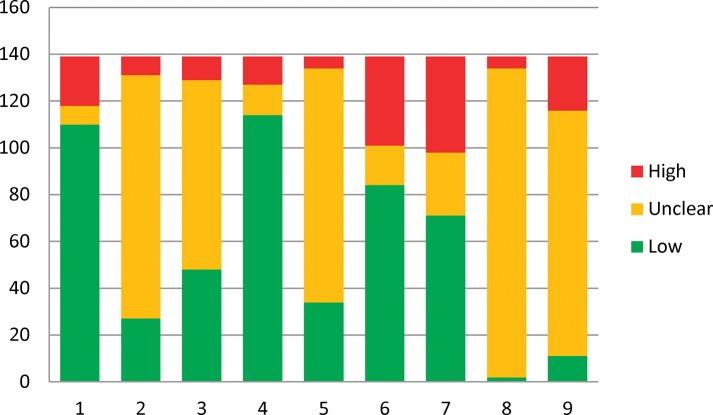
Summary of the quality assessment of the 139 cross-over trials 1. Appropriate cross-over design; 2. Randomized order of receiving treatment; 3. Carry-over effects; 4. Unbiased data; 5. Allocation concealment; 6. Blinding; 7. Incomplete outcome data; 8. Selective outcome reporting; 9. Other bias

The summaries of each item considered in study quality assessment are detailed in Table 2 and Fig 2. The majority of cross-over designs (110, 79.1%) were considered to be at a low risk of bias. Short washout period was the primary cause of high risk of bias. As for the randomized order of receiving treatments, only 27 (19.4%) studies were considered as low risk of bias as the method for randomization were adequate and reported clearly. Most studies however (74.8%) did not report the methods for sequence generation.

**Table 2:** The results of quality reassessment of different items.

Risk of bias	Appropriate cross-over design	Randomized order of receiving treatments	Carry-over effects	Unbiased data	Allocation concealment	Blinding	Incomplete outcome data	Selective outcome reporting	Other bias
**Low N(%)**	110(79.1%)	27(19.4%)	48(34.5%)	114(82.0%)	34(24.5%)	84(60.4%)	71(51.1%)	2(1.4%)	11(7.9%)
**Unclear N(%)**	8(5.8%)	104(74.8%)	81(58.3%)	13(9.4%)	100(71.9%)	17(12.2%)	27(19.4%)	132(95.0%)	105(75.5%)
**High N(%)**	21(15.1%)	8(5.8%)	10(7.2%)	12(8.6%)	5(3.6%)	38(27.3%)	41(29.5%)	5(3.6%)	23(16.5%)

The risk of bias from carry-over effects was considered as low in 48 (34.5%) studies but the majority (81, 58.3%) did not evaluate the carry-over effects. Study outcomes were reported at every stage in 114 (82.0%) studies and were therefore judged as low risk of bias for reporting of findings.

As for allocation concealment, only 34(24.5%) studies were judged to have a low risk of bias. Most studies did not describe the approaches to ensure the allocation sequence was concealed. Additionally, 84 (60.4%) studies had a low risk of bias in items of blinding.

Approximately half of the included studies (51.1%) had a low risk of bias from incomplete outcome data reporting. Insufficient information was reported for the judgment of selective outcome reporting in most studies. Only 2 (1.4%) studies were considered to have low risk of bias. Similarly, very few studies (11, 7.9%) were considered to have low risk on other biases due to insufficient information being reported to allow judgment.

## Discussion

Cross-over studies constitute a substantial proportion of the original studies involved in systematic reviews. According to our study, by 2013, a total of 5495 CSRs had been published in the Cochrane Library, and 688 (12.52%) of them included one or more cross-over study. One review reported that 8% of all the trials registered in the Cochrane Controlled Register (the January 2001 issue of the Cochrane Library)were cross-over studies and 18% of CSRs referred to cross-over studies [16].

Despite being widely included in CSRs, cross-over study quality was not assessed comprehensively and correctly by most review authors. Our study indicated that the evaluation of risk of bias in cross-over design, carry-over effects, and unbiased data reporting was almost always neglected in current CSRs. It has been suggested that cross-over design is suitable to study short acting or temporary relief interventions for stable conditions [3,5]. Inappropriate application of a cross-over design can lead to erroneous conclusions. Carry-over is the persistence of an effect in a subsequent period of treatment due to treatment in a previous period [1,3,5]. It is measurable [1,17,18] and suggested to be the major drawback for cross-over trials [3]. Our study revealed that most of the included cross-over trials did not evaluate or discuss the carry-over effect. A previous review of 116 cross-over trials showed that carry-over effects were addressed in 29% of all the trials [19], which is a little lower than our results. This slightly lower proportion, compared to our findings, may be because we only included cross-over studies from CSRs and some low quality cross-over studies may have been excluded by the review authors. As already stated above, cross-over design has the strength of comparing treatments within individuals. When studies report results for the first period of treatment only, it removes this strength, and bias therefore exists in so-called “two stage analysis” where findings are reported separately depending on the order of treatment allocation[20].

Our re-evaluation of study biases indicates that previous CSRs had incompletely assessed risk of bias or study quality. This may be having resulted in biased or incorrect assessment of the quality of data included in systematic reviews. Factors including the risk of bias in the carry-over effects, appropriate cross-over design and unbiased data existed to different degrees in cross-over designs but were neglected in assessment of study quality. The implication here is that studies judged as low risk of bias in CSRs may in fact be classed as high risk of bias in some domains and this change could affect the overall quality level judged for included in different reviews.

Although it was not a main objective for the present study, the reassessment results provide an overview of the quality of cross-over studies included in CSRs generally. The overall quality of current cross-over studies was moderate, with high quality cross-over studies being rare. Furthermore, this study also indicated that biases for cross-over studies mainly arise from incomplete outcome data, blinding, and inappropriate cross-over design.

To the best of our knowledge, this is the first and most comprehensive study evaluating the quality of cross-over trials that have been included in CSRs. We summarized a nine-item check list for the evaluation of cross-over trials and this can be used for future systematic reviews. Our experience in using this check list suggested it is explicit and easy to implement. The main limitation of this study is that we only assessed studies included in CSRs and it remains unclear whether the quality assessment of cross-over studies included in non-CSRs is similar to that in CSRs or not. However, we speculate that the assessment of cross-over study quality is likely to be less consistent in other reviews because CSRs preparations is subject to strict criteria and is supported by the Cochrane review group [21, 22].

This study has important implications for future research. Firstly, we provide a simple and practical 9-items checklist for the quality assessment of the cross-over studies, which can easily be adopted in future reviews. In addition, this study provides a preliminary evaluation of study quality of cross-over studies and indicated the general strength and weakness. These results will hopefully guide the study design and improve the reporting of methods and results in future cross-over studies.

In conclusion, the current quality assessment standards are not adequate to assess cross-over studies. Items specific to cross-over trials, leading to potential risk of bias are generally neglected in the quality assessment of CSRs. A proposed check list for the evaluation of cross-over trials quality in future review is provided.

## Supporting Information

S1 PRISMA Checklist PRISMA 2009 checklist for the manuscript. (DOC)

S1 Appendix References of 60 included Cochrane Systematic Reviews. (DOC)

## References

[1] Senn SJ. Cross-over trials in clinical research Chichester: John Wiley 2002; 1.

[2] Maclure M. The case-crossover design: a method for studying transient effects on the risk of acute events. Am J Epidemiol. 1991; 133(2): 144–153. 1985444 10.1093/oxfordjournals.aje.a115853

[3] The Cochrane Collaboration. Cochrane handbook for systematic reviews of interventions, version 5.0.2. Available: www.cochrane-handbook.org. 2008; Accessed 29 May 2010.

[4] Cleophas TJ, De Vogel EM. Crossover studies are a better format for comparing equivalent treatments than parallel-group studies. Pharm World Sci. 1998; 20(3): 113–117. 9618734 10.1023/a:1008626002664

[5] Brown BW Jr. The crossover experiment for clinical trials. Biometrics. 1980; 36: 69–79. 7370374

[6] Virgili G, Acosta R, Grover LL, Bentley SA, Giacomelli G. Reading aids for adults with low vision. Cochrane Database Syst Rev. 2006; 18 (4):CD003303. 10.1002/14651858.CD003303.pub3 PMC4288929 24154864

[7] Herbert RD, de Noronha M, Kamper SJ. Stretching to prevent or reduce muscle soreness after exercise. Cochrane Database Syst Rev. 2007; 17 (4):CD004577. 10.1002/14651858.CD004577.pub2 17943822

[8] French SD, Cameron M, Walker BF, Reggars JW, Esterman AJ. Superficial heat or cold for low back pain. Cochrane Database Syst Rev. 2006; (1): CD004750. 10.1002/14651858.CD004750.pub2 PMC8846312 16437495

[9] Lemyre B, Davis PG, de Paoli AG. Nasal intermittent positive pressure ventilation (NIPPV) versus nasal continuous positive airway pressure (NCPAP) for apnea of prematurity. Cochrane Database Syst Rev. 2002; (1): CD002272. 10.1002/14651858.CD002272 PMC13501246 11869635

[10] Pringsheim T, Marras C. Pimozide for tics in Toilette’s syndrome. Cochrane Database Syst Rev. 2009;(2): CD006996. 10.1002/14651858.CD006996.pub2 PMC7212051 19370666

[11] Evans DJ, Cullinan P, Geddes DM. Cyclosporin as an oral corticosteroid sparing agent in stable asthma. Cochrane Database Syst Rev. 2001; (2):CD002993. 10.1002/14651858.CD002993 PMC8407285 11406057

[12] Moja PL, Cusi C, Sterzi RR, Canepari C. Selective serotonin re-uptake inhibitors (SSRIs) for preventing migraine and tension-type headaches. Cochrane Database Syst Rev. 2005; 20 (3):CD002919. 10.1002/14651858.CD002919.pub2 16034880

[13] Blackhall K, Appleton S, Cates CJ. Ionisers for chronic asthma. Cochrane Database Syst Rev. 2003; (3):CD002986. 10.1002/14651858.CD002986 12917939

[14] McKean M, Ducharme F. Inhaled steroids for episodic viral wheeze of childhood. Cochrane Database Syst Rev. 2000; (2):CD001107. 10.1002/14651858.CD001107 PMC8406470 10796596

[15] Davies AN, Shorthose K. Parasympathomimetic drugs for the treatment of salivary gland dysfunction due to radiotherapy. Cochrane Database Syst Rev. 2007; 18 (3):CD003782. 10.1002/14651858.CD003782.pub2 17636736

[16] Elbourne DR, Altman DG, Higgins JP, Curtin F, Worthington HV, Vail A. Meta-analyses involving cross-over trials: methodological issues. Int J Epid. 2002; 31: 140–149. 10.1093/ije/31.1.140 11914310

[17] Senn SJ. Statistical issues in drug development Chichester: John Wiley 1993; xx p: 273–284

[18] Cleophas TJ. A simple method for the estimation of interaction bias in crossover studies. J Clin Pharmacol. 1990; 30: 1036–1040. 2243151 10.1002/j.1552-4604.1990.tb03591.x

[19] Mills EJ, Chan AW, Wu P, Vail A, Guyatt GH, Altman DG. Design, analysis, and presentation of crossover trials. Trials. 2009; 30(10): 27. 10.1186/1745-6215-10-27 PMC2683810 19405975

[20] Freeman PR. The performance of the two-stage analysis of two-treatment, two-period cross-over trials. Stat Med. 1989; 8: 1421–1432. 2616932 10.1002/sim.4780081202

[21] Bero L, Rennie D. The Cochrane Collaboration. Preparing, maintaining, and disseminating systematic reviews of the effects of health care. JAMA. 1995; 74: 1935–1938. 10.1001/jama.274.24.1935 8568988

[22] Clarke M. The Cochrane Collaboration and systematic reviews. Br J Surg. 2007; 94(4): 391–392. 17380545 10.1002/bjs.5812

