# Supplementary material for: The Method Quality of Cross-Over Studies Involved in Cochrane Systematic Reviews
Source: PLoS One. 2015 Apr 13;10(4):e0120519. doi: 10.1371/journal.pone.0120519 (PMC4395015; doi:10.1371/journal.pone.0120519)
Supplement: S1 Appendix — (DOC) [file pone.0120519.s002.doc]

**S1_Appendix**：**References of 60 included Cochrane Systematic Reviews.**

1. Wang Y, Pan T, Wang Q and Guo Z (2009) Additional bedtime H2-receptor antagonist for the control of nocturnal gastric acid breakthrough. The Cochrane database of systematic reviews: CD004275.
2. Alhasso AA, McKinlay J, Patrick K and Stewart L (2006) Anticholinergic drugs versus non-drug active therapies for overactive bladder syndrome in adults. The Cochrane database of systematic reviews: CD003193.
3. Vignatelli L, D'Alessandro R and Candelise L (2008) Antidepressant drugs for narcolepsy. The Cochrane database of systematic reviews: CD003724.
4. Bjelakovic G, Nikolova D, Gluud LL, Simonetti RG and Gluud C (2012) Antioxidant supplements for prevention of mortality in healthy participants and patients with various diseases. The Cochrane database of systematic reviews: CD007176.
5. Seidel S, Aigner M, Ossege M, Pernicka E, Wildner B, et al. (2008) Antipsychotics for acute and chronic pain in adults. The Cochrane database of systematic reviews: CD004844.
6. Crosby NJ, Deane KH and Clarke CE (2003) Beta-blocker therapy for tremor in Parkinson's disease. The Cochrane database of systematic reviews: CD003361.
7. Quartero AO, Meineche-Schmidt V, Muris J, Rubin G and de Wit N (2005) Bulking agents, antispasmodic and antidepressant medication for the treatment of irritable bowel syndrome. The Cochrane database of systematic reviews: CD003460.
8. Bara AI and Barley EA (2001) Caffeine for asthma. The Cochrane database of systematic reviews: CD001112.
9. Rerkasem K and Rothwell PM (2011) Carotid endarterectomy for symptomatic carotid stenosis. The Cochrane database of systematic reviews: CD001081.
10. Dean T, Dewey A, Bara A, Lasserson TJ and Walters EH (2003) Chloroquine as a steroid sparing agent for asthma. The Cochrane database of systematic reviews: CD003275.
11. Gray PH and Flenady V (2011) Cot-nursing versus incubator care for preterm infants. The Cochrane database of systematic reviews: CD003062.
12. Pope J, Fenlon D, Thompson A, Shea B, Furst D, et al. (2000) Cyclofenil for Raynaud's phenomenon in progressive systemic sclerosis. The Cochrane database of systematic reviews: CD000955.
13. Evans DJ, Cullinan P and Geddes DM (2001) Cyclosporin as an oral corticosteroid sparing agent in stable asthma. The Cochrane database of systematic reviews: CD002993.
14. Fioravanti M and Yanagi M (2005) Cytidinediphosphocholine (CDP-choline) for cognitive and behavioural disturbances associated with chronic cerebral disorders in the elderly. The Cochrane database of systematic reviews: CD000269.
15. Roberts DJ, Rees D, Howard J, Hyde C, Alderson P, et al. (2005) Desferrioxamine mesylate for managing transfusional iron overload in people with transfusion-dependent thalassaemia. The Cochrane database of systematic reviews: CD004450.
16. Adams NP, Bestall JC, Jones P, Lasserson TJ, Griffiths B, et al. (2008) Fluticasone at different doses for chronic asthma in adults and children. The Cochrane database of systematic reviews: CD003534.
17. Lonergan E, Luxenberg J and Colford J (2002) Haloperidol for agitation in dementia. The Cochrane database of systematic reviews: CD002852.
18. Klenoff-Brumberg HL and Genen LH (2003) High versus low medium chain triglyceride content of formula for promoting short term growth of preterm infants. The Cochrane database of systematic reviews: CD002777.
19. Koh MS, Tee A, Lasserson TJ and Irving LB (2007) Inhaled corticosteroids compared to placebo for prevention of exercise induced bronchoconstriction. The Cochrane database of systematic reviews: CD002739.
20. McKean M and Ducharme F (2000) Inhaled steroids for episodic viral wheeze of childhood. The Cochrane database of systematic reviews: CD001107.
21. Miller D, Livingstone V and Herbison P (2008) Interventions for relieving the pain and discomfort of screening mammography. The Cochrane database of systematic reviews: CD002942.
22. Blackhall K, Appleton S and Cates CJ (2003) Ionisers for chronic asthma. The Cochrane database of systematic reviews: CD002986.
23. Schwarzer G, Bassler D, Mitra A, Ducharme FM and Forster J (2004) Ketotifen alone or as additional medication for long-term control of asthma and wheeze in children. The Cochrane database of systematic reviews: CD001384.
24. Osborn DA and Henderson-Smart DJ (2000) Kinesthetic stimulation for treating apnea in preterm infants. The Cochrane database of systematic reviews: CD000499.
25. Tjia-Leong E, Leong K and Marson AG (2010) Lamotrigine adjunctive therapy for refractory generalized tonic-clonic seizures. The Cochrane database of systematic reviews: CD007783.
26. Tuunainen A, Kripke DF and Endo T (2004) Light therapy for non-seasonal depression. The Cochrane database of systematic reviews: CD004050.
27. Richeldi L, Ferrara G, Fabbri LM, Lasserson TJ and Gibson PG (2005) Macrolides for chronic asthma. The Cochrane database of systematic reviews: CD002997.
28. Garrison SR, Allan GM, Sekhon RK, Musini VM and Khan KM (2012) Magnesium for skeletal muscle cramps. The Cochrane database of systematic reviews 9: CD009402.
29. Schmidt HM, Hagen M, Kriston L, Soares-Weiser K, Maayan N, et al. (2012) Management of sexual dysfunction due to antipsychotic drug therapy. The Cochrane database of systematic reviews 11: CD003546.
30. Hondras MA, Linde K and Jones AP (2005) Manual therapy for asthma. The Cochrane database of systematic reviews: CD001002.
31. Florcruz NV and Peczon I, Jr. (2008) Medical interventions for fungal keratitis. The Cochrane database of systematic reviews: CD004241.
32. Tusting LS, Thwing J, Sinclair D, Fillinger U, Gimnig J, et al. (2013) Mosquito larval source management for controlling malaria. The Cochrane database of systematic reviews 8: CD008923.
33. Cepeda MS, Carr DB, Lau J and Alvarez H (2006) Music for pain relief. The Cochrane database of systematic reviews: CD004843.
34. Bradt J and Dileo C (2009) Music for stress and anxiety reduction in coronary heart disease patients. The Cochrane database of systematic reviews: CD006577.
35. Vink AC, Birks JS, Bruinsma MS and Scholten RJ (2004) Music therapy for people with dementia. The Cochrane database of systematic reviews: CD003477.
36. Lemyre B, Davis PG and de Paoli AG (2002) Nasal intermittent positive pressure ventilation (NIPPV) versus nasal continuous positive airway pressure (NCPAP) for apnea of prematurity. The Cochrane database of systematic reviews: CD002272.
37. Nelson R (2006) Non surgical therapy for anal fissure. The Cochrane database of systematic reviews: CD003431.
38. Cody JD, Richardson K, Moehrer B, Hextall A and Glazener CM (2009) Oestrogen therapy for urinary incontinence in post-menopausal women. The Cochrane database of systematic reviews: CD001405.
39. Nuesch E, Rutjes AW, Husni E, Welch V and Juni P (2009) Oral or transdermal opioids for osteoarthritis of the knee or hip. The Cochrane database of systematic reviews: CD003115.
40. Seddon P, Bara A, Ducharme FM and Lasserson TJ (2006) Oral xanthines as maintenance treatment for asthma in children. The Cochrane database of systematic reviews: CD002885.
41. Davies AN and Shorthose K (2007) Parasympathomimetic drugs for the treatment of salivary gland dysfunction due to radiotherapy. The Cochrane database of systematic reviews: CD003782.
42. Fleminger S, Greenwood RJ and Oliver DL (2006) Pharmacological management for agitation and aggression in people with acquired brain injury. The Cochrane database of systematic reviews: CD003299.
43. Brookes G and Ahmed AG (2006) Pharmacological treatments for psychosis-related polydipsia. The Cochrane database of systematic reviews: CD003544.
44. Gois PH and Souza ER (2013) Pharmacotherapy for hyperuricemia in hypertensive patients. The Cochrane database of systematic reviews 1: CD008652.
45. Tomlinson CL, Patel S, Meek C, Herd CP, Clarke CE, et al. (2013) Physiotherapy versus placebo or no intervention in Parkinson's disease. The Cochrane database of systematic reviews 9: CD002817.
46. Cordeiro NJ and Oniyangi O (2004) Phytomedicines (medicines derived from plants) for sickle cell disease. The Cochrane database of systematic reviews: CD004448.
47. Pringsheim T and Marras C (2009) Pimozide for tics in Tourette's syndrome. The Cochrane database of systematic reviews: CD006996.
48. Carrick S, Ghersi D, Wilcken N and Simes J (2004) Platinum containing regimens for metastatic breast cancer. The Cochrane database of systematic reviews: CD003374.
49. Davies EG, Riddington C, Lottenberg R and Dower N (2004) Pneumococcal vaccines for sickle cell disease. The Cochrane database of systematic reviews: CD003885.
50. Moja PL, Cusi C, Sterzi RR and Canepari C (2005) Selective serotonin re-uptake inhibitors (SSRIs) for preventing migraine and tension-type headaches. The Cochrane database of systematic reviews: CD002919.
51. Siebenhofer A, Plank J, Berghold A, Jeitler K, Horvath K, et al. (2006) Short acting insulin analogues versus regular human insulin in patients with diabetes mellitus. The Cochrane database of systematic reviews: CD003287.
52. Herbert RD and de Noronha M (2007) Stretching to prevent or reduce muscle soreness after exercise. The Cochrane database of systematic reviews: CD004577.
53. French SD, Cameron M, Walker BF, Reggars JW and Esterman AJ (2006) Superficial heat or cold for low back pain. The Cochrane database of systematic reviews: CD004750.
54. Yong SL, Coulthard P and Wrzosek A (2012) Supplemental perioperative steroids for surgical patients with adrenal insufficiency. The Cochrane database of systematic reviews 12: CD005367.
55. Ghersi D, Wilcken N, Simes J and Donoghue E (2005) Taxane containing regimens for metastatic breast cancer. The Cochrane database of systematic reviews: CD003366.
56. Aziz NA, Leonardi-Bee J, Phillips M, Gladman JR, Legg L, et al. (2008) Therapy-based rehabilitation services for patients living at home more than one year after stroke. The Cochrane database of systematic reviews: CD005952.
57. Derry S, Lloyd R, Moore RA and McQuay HJ (2009) Topical capsaicin for chronic neuropathic pain in adults. The Cochrane database of systematic reviews: CD007393.
58. Robb KA, Bennett MI, Johnson MI, Simpson KJ and Oxberry SG (2008) Transcutaneous electric nerve stimulation (TENS) for cancer pain in adults. The Cochrane database of systematic reviews: CD006276.
59. Palmer SC, McGregor DO, Craig JC, Elder G, Macaskill P, et al. (2009) Vitamin D compounds for people with chronic kidney disease not requiring dialysis. The Cochrane database of systematic reviews: CD008175.
60. Kelly SA, Summerbell CD, Brynes A, Whittaker V and Frost G (2007) Wholegrain cereals for coronary heart disease. The Cochrane database of systematic reviews: CD005051.
